# Supplementary material for: Chromothripsis during telomere crisis is independent of NHEJ, and consistent with a replicative origin
Source: Genome Res. 2019 May;29(5):737–49. doi: 10.1101/gr.240705.118 (PMC6499312; doi:10.1101/gr.240705.118)
Supplement: Supplemental Material [file supp_gr.240705.118_Supplemental_file_1.zip › contigs/annotated_contigs/DB95/contig.2.DB95_length_373_mean_cov_5.3163538874.docx]

**DB95_length_373_mean_cov_5.3163538874**

```
GTTTTCCCAGAGCAGTGCCTCTCCATAACTCTTCCTAGGGCCCGTCTAATCCTGACACTGCAGGT|AGAATTCTAGGTGCAAG|CTACC
 >chrX:138384829-138384911 + E=7e-38 p=0e+00                      >chrX:138385220-13838
CAAAGGCTGCCATTTTGAGATGATTCTGCCCCCACCAGTTTCTCCAGGCCAGAAGCTGATTGTCTGTTGTTTTAGGTGCAGCTCAAAAC
5526 + E=4e-170
CTCCAGTTTTGCCTTCTGTAAGAGCACTGACCCAACTCTGCAAACCATTGCTAGTTAGTAACAATCAGAAAAGGATATGTGAATGCTTA

TTCTTCTCCATGCTATTTTCTAACCTGGCTGTATTAACAGCTAATCAGAAATTAATGCTATGAGTAGGCACTCTTCTGACTTAAGAGGG

AGCAAGTCAGCGAAATA|CT
```
